# The anti-COVID-19 drug Paxlovid crosses biological barriers of the placenta and brain in rats

**DOI:** 10.1038/s44298-023-00013-1

**Published:** 2024-01-24

**Authors:** Wan-Hsin Lee, Chung-Kai Sun, Chun-Hao Chang, Muh-Hwa Yang, Tung-Hu Tsai

**Affiliations:** 1 Institute of Traditional Medicine, College of Medicine, National Yang Ming Chiao Tung University, Taipei, 112 Taiwan; 2 Institute of Clinical Medicine, College of Medicine, National Yang Ming Chiao Tung University, Taipei, 112 Taiwan; 3 Graduate Institute of Acupuncture Science, China Medical University, Taichung, 404 Taiwan; 4 Department of Chemistry, National Sun Yat-Sen University, Kaohsiung, 804 Taiwan; 5 School of Pharmacy, Kaohsiung Medical University, Kaohsiung, 807 Taiwan

**Keywords:** Drug discovery, Medical research

## Abstract

Paxlovid (nirmatrelvir plus ritonavir) is an orally available drug for the treatment of COVID-19 disease. However, limited information remains on the biological barrier transfer of nirmatrelvir. In the present study, we investigated whether nirmatrelvir crosses the blood-placenta barrier to reach the conceptus (the collective term for the fetus, placenta, and amniotic fluid) during pregnancy and the blood–brain barrier (BBB) in male rats. Additionally, in this study, gender and pregnancy issues were investigated. Multisite microdialysis coupled with validated UHPLC-MS/MS was developed to monitor nirmatrelvir levels in maternal blood and the conceptus in pregnant rats and of the blood and brain in male and nonpregnant female rats after administration of nirmatrelvir (15 mg/kg, i.v.) alone and nirmatrelvir (15 mg/kg, i.v.) combined with ritonavir (7 mg/kg, i.v.). Pharmacokinetic results showed that nirmatrelvir rapidly penetrates the blood–placenta barrier to reach the conceptus after administration of nirmatrelvir (15 mg/kg, i.v.) alone and nirmatrelvir (15 mg/kg, i.v.) combined with ritonavir (7 mg/kg, i.v.) in pregnant rats. Nirmatrelvir also crosses the BBB in male and nonpregnant female rats in the same dose regimen. Compared to sex and pregnancy factors, the results show that protein-unbound nirmatrelvir increased significantly during pregnancy and did not differ between nonpregnant female and male rats. The results indicated that the concentrations of nirmatrelvir in the blood, conceptus, and brain were higher than the effective concentration of 90% (total EC_90_ = 292 ng/mL, unbound EC_90_ = 90.5 ng/mL, 181 nM) after the administration of nirmatrelvir plus ritonavir. Ritonavir provides a synergistic pharmacokinetic effect. Pregnancy is an important issue with increased protein-unbound nirmatrelvir in the blood and tissues.

## Introduction

Coronavirus disease 2019 (COVID-19) is a serious infectious disease that has ravaged the world in the past 3 years^1^. Although effective vaccines^2,3^ and antiviral drugs have been made available with emergency use authorization (EUA) to patients at high risk of severe COVID-19, symptoms and complications continues to cause many deaths worldwide^4,5^. Furthermore, on 22 December 2021, Paxlovid (nirmatrelvir plus ritonavir), developed by Pfizer^6–8^, received EUA from the FDA for the treatment of patients with COVID-19^9^. Nirmatrelvir (PF-07321332) is a new oral antiviral drug that blocks the replication of the severe acute respiratory syndrome coronavirus 2 (SARS-CoV-2) virus by inhibiting the active site of the main protease (Mpro)^10,11^. At the recommended dose for treatment to reach an effective concentration of 90% (total EC_90_ = 292 ng/mL, unbound EC_90_ = 90.5 ng/mL, 181 nM), Pfizer observed an effective recommended dose in clinical trials, which is 300 mg of nirmatrelvir (administered as two 150 mg tablets) with 100 mg of ritonavir (one 100 mg tablet)^10,12^, while ritonavir, a cytochrome P450 (CYP)3A4 inhibitor, is an antiretroviral drug used to treat human immunodeficiency virus type 1 (HIV/AIDS)^13^.

Due to the emergency status of the disease, clinical data may not yet be sufficient to support the treatment of COVID-19 with antiviral drugs, such as nirmatrelvir, molnupiravir and its metabolite NHC (in full), in pregnant and breastfeeding women^14,15^. Recently, we have shown that molnupiravir and its main metabolite traverse the placental barrier in pregnant rats^16^ and exhibit the potential to cross the blood–brain barrier (BBB) in male rats^17^. This study aimed to extend this evaluation to the antiviral drug Paxlovid for COVID-19, assessing its biological barrier penetration through the placenta and BBB. In addition, sex and pregnancy status were investigated.

Recent studies have investigated the pharmacokinetics (PK) of Paxlovid in pregnant women^18^, providing insights into its safety and effectiveness for both the pregnant individuals and the fetus in clinical usage. Pregnant women exhibit physiological changes^19^, such as changes in the immune and cardiopulmonary systems, which can increase the chance of serious illness in pregnant women after infection^20^. For these reasons, the option of using nirmatrelvir may be safer than using molnupiravir as an orally administered treatment of COVID-19 in pregnant women. However, the specific target concentrations of nirmatrelvir should be required in the maternal environment and conceptus (the collective term for the fetus, placenta, and amniotic fluid) to support further pharmacological and toxicological studies.

Previous literature demonstrated that SARS-CoV-2 uses angiotensin-converting enzyme 2 (ACE2) to enter target cells, making them potential infection targets for SARS-CoV-2^21,22^. Although the lungs are the main site of infection with SARS-CoV-2, the brain is also infected and can cause neurological sequelae. The SARS-CoV-2 virus has been shown to exist in the cerebrospinal fluid of humans^23^, and the neuroinvasive potential of this virus cannot be ignored^24^. The virus may reach the brain through the systemic circulation or the upper nasal transcriptional pathway and attack the nervous system^21,25^. Therefore, whether antiviral drugs can reach the brain is the current research target for patients with COVID-19 complicated by brain infection.

Microdialysis is a continuous sampling technique that consists of a semipermeable hollow fiber membrane at its tip that is used to collect analytes of unbound protein in extracellular fluids. The combined model of microdialysis and ultrahigh-performance liquid chromatography coupled to tandem mass spectrometry (UHPLC‒MS/MS) was developed to collect blood and conceptus samples from pregnant rats^26^ and brain^27^ from male rats.

Our hypothesis is that nirmatrelvir may cross biological barriers to reach an effective concentration and pharmacokinetic synergistic effect occurring in nirmatrelvir plus ritonavir. Pregnancy factors may affect the pharmacokinetics of nirmatrelvir. To investigate these hypotheses, a multisite microdialysis sampling system coupled with a UHPLC‒MS/MS analytical system was developed to investigate the pharmacokinetic synergistic effect on Paxlovid in pregnant, nonpregnant female and male rats.

## Materials and methods

### Chemicals and reagents

Nirmatrelvir was obtained from MedChemExpress (Monmouth Junction, NJ, USA). Ritonavir was purchased from Cayman Chemical Company (Ann Arbor, Michigan, USA). Dimethyl sulfoxide (DMSO), polyethylene glycol 400 (PEG400), heparin sodium salt, and urethane were purchased from Sigma‒Aldrich Chemicals (St. Louis, MO, USA). Ethanol was acquired from ECHO Chemical Co., Ltd. (Miaoli County, Taiwan). Acetonitrile (mass grade) was acquired from J.T. Baker, Inc. (Phillipsburg, NJ). Triple deionized water from Millipore (Bedford, MA, USA) was used for all aqueous samples and mobile phase preparation. A standard stock solution of nirmatrelvir (1 mg/mL) was dissolved in ethanol and then stored at −20 °C for experimental use.

### UHPLC‒MS/MS conditions

The UHPLC‒MS/MS system (Shimadzu LC‒MS/MS 8030, Kyoto, Japan) used an Inertsil ODS-3 HP column (100 × 2.1 mm, particle size 3 μm, GL sciences, Japan) operated in a column oven at approximately 35 °C for the entire experimental analysis. The UHPLC‒MS/MS system consisted of a quadrupole mass spectrometer equipped with a positive mode electrospray ionization (ESI+) source that was used for nirmatrelvir analysis. Mobile phases A and B consisted of 0.1% formic acid in water (pH = 2.6) and acetonitrile with isocratic elution at 40:60 (v/v), with a flow rate of 0.4 mL/min and an injection volume of 5 μL. The mass spectrometric conditions were set as follows: desolvation line temperature, 250 °C; heat temperature, 400 °C; interface voltage, 3.5 kV; nebulizing gas flow, 3.0 L/min; and drying gas flow, 15 L/min. Detection and quantification were performed using multiple reaction monitoring (MRM) for the parent-to-product ion transitions m/z 500.30 → 319.10 for nirmatrelvir (Supplementary Fig. 1).

### Microdialysis experiment

The microdialysis instrument consisted of a microinjection pump (CMA/400; Solna, Sweden) and a microfraction collector (CMA/142). The laboratory-made microdialysis probe consisted of a concentric silica capillary and a semipermeable dialysis membrane (Spectrum, New Brunswick, NJ, USA) with a fiber diameter of 200 μm and a molecular weight cutoff of 13 kDa. The active lengths of the blood probe were 1.1 cm, and those of the conceptus and brain probes were 0.6 cm. The microinjection pump contained the perfusion fluid, which consisted of 3.5 mM citric acid, 7.5 mM sodium citrate, and 13.6 mM D-(+)-glucose (acid citrate dextrose; ACD solution), which was continuously infused at a flow rate of 2 μL/min; dialysates were collected every 20 min by a microfraction collector for 6 h, and then the samples were stored at −20 °C for analysis.

### Experimental animals

Sprague‒Dawley rats (pregnant female, 16 days of gestation, 315 ± 20 g), adult male Sprague‒Dawley rats (6 weeks old, weighing 200 ± 20 g) and adult female Sprague‒Dawley rats (6 weeks old, weighing 200 ± 20 g) were purchased from the Laboratory Animal Center at National Yang Ming Chiao Tung University (Taipei, Taiwan). Animal experiments in this study were approved by the Institutional Animal Care and Use Committee of the National Yang Ming Chiao Tung University (IACUC no. 1110605, 1110606). The guidelines for animal surgical procedures followed the Guide for the Care and Use of Laboratory Animals^28^.

### Pregnant female

Pregnant rats were anesthetized with urethane (1 g/kg, i.p.). After confirming that the rat was completely anesthetized, a polyethylene PE 50 tube (Becton Dickinson; Sparks, MD, USA) was first inserted into the left femoral vein for drug administration. A blood microdialysis probe was inserted into the left jugular vein for the collection of maternal blood dialysate samples. When the blood microdialysis probe was in place, open abdominal surgery was performed, and microdialysis probes were placed in the three tissues of the fetus, placenta, and amniotic fluid for conceptus dialysate sample collection. According to the literature, it is known that there is species specificity in the uterine cervix and uterine structure among mammals^29^. In rats, for instance, the fertilized egg implants and develops in the uterine horn. During our PK experiments, we randomly select three healthy embryos from both sides of the uterine horns. The guidelines for the animal surgical procedure followed the Guide for the Care and Use of Laboratory Animals and ARRIVE (Animal Research: Reporting of In Vivo Experiments) guidelines^30^.

### Male and nonpregnant female rats

Following complete anesthesia, PE 50 (polyethylene tube, Becton Dickinson, MD, USA) was embedded in the left femoral vein to facilitate drug administration. Once the blood microdialysis probe was placed, the animal was placed on a stereotaxic instrument to facilitate brain surgery. The surgical procedure involved marking the location of the striatum at +0.2 mm anteroposterior, +3.2 mm mediolateral, and −7.5 mm dorsoventral to the bregma, followed by drilling a hole with a pen drill to place the brain microdialysis probe.

### Drug administration

The recommended dose of Paxlovid is 300 mg of nirmatrelvir plus 100 mg of ritonavir for humans. Using a reference, the recommended doses for humans were converted to equivalent doses in rats^31^. The drug was originally designed to be administered orally. To avoid disturbance by the first-pass effect, intravenous administration was used in the experiment, and bioavailability was corrected to identify the dose administered. The bioavailability of nirmatrelvir and ritonavir is approximately 50%^32^ and 80%^33^, respectively. Nirmatrelvir was dissolved in 10% DMSO and 45% PEG 400 with normal saline for nirmatrelvir administration (15 mg/kg, i.v.), and Paxlovid of nirmatrelvir (15 mg/kg, i.v.) plus ritonavir (7 mg/kg, i.v.) was administered after the microdialysis probe implantation.

### Statistical analysis

The analysis of the data of the pharmacokinetic parameters was performed with WinNonlin Standard Edition software (version 1.0, Pharsight Corp., Mountain View, CA, USA). The pharmacokinetic curves were generated using SigmaPlot software (version 12.0, Systat Software Inc., San Jose, CA). The total pharmacokinetic parameters were analyzed by one-way analysis of variance (ANOVA) with Tukey’s post hoc Honest Significant Difference (HSD) test and an independent *t* test. One-way analysis of variance (ANOVA) with Tukey’s post hoc HSD test was used to compare the values obtained from different tissues between the two groups; the independent *t* test was used to analyze the effects of ritonavir in the same tissue. Post hoc power analysis was used to determine experimental group size and number. The results with a *p* value < 0.05 were considered statistically significant. Data are presented as the mean ± standard error mean (S.E.M.; *n* = 6).

## Results

### Optimized UHPLC‒MS/MS conditions

**Fig. 1:**
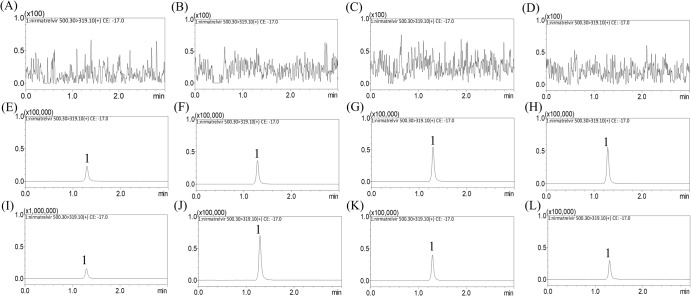
MRM chromatograms of nirmatrelvir in maternal blood and conceptus of pregnant rats. Representative MRM chromatograms of the experiments. **A**–**D** show blank dialysates from **A** maternal blood, **B** placenta, **C** fetus, and **D** amniotic fluid; **E**–**H** are blank dialysates spiked with nirmatrelvir (50 ng/mL) of **E** maternal blood, **F** placenta, **G** fetus, and **H** amniotic fluid; **I**–**L** are experimental samples collected at 60 min after nirmatrelvir administration (15 mg/kg, i.v.), from **I** maternal blood containing nirmatrelvir (344.8 ng/mL), **J** placenta containing nirmatrelvir (95.13 ng/mL), **K** fetus containing nirmatrelvir (37 ng/mL), and **L** amniotic fluid containing nirmatrelvir (27.61 ng/mL); peak 1 = nirmatrelvir.

**Fig. 2:**
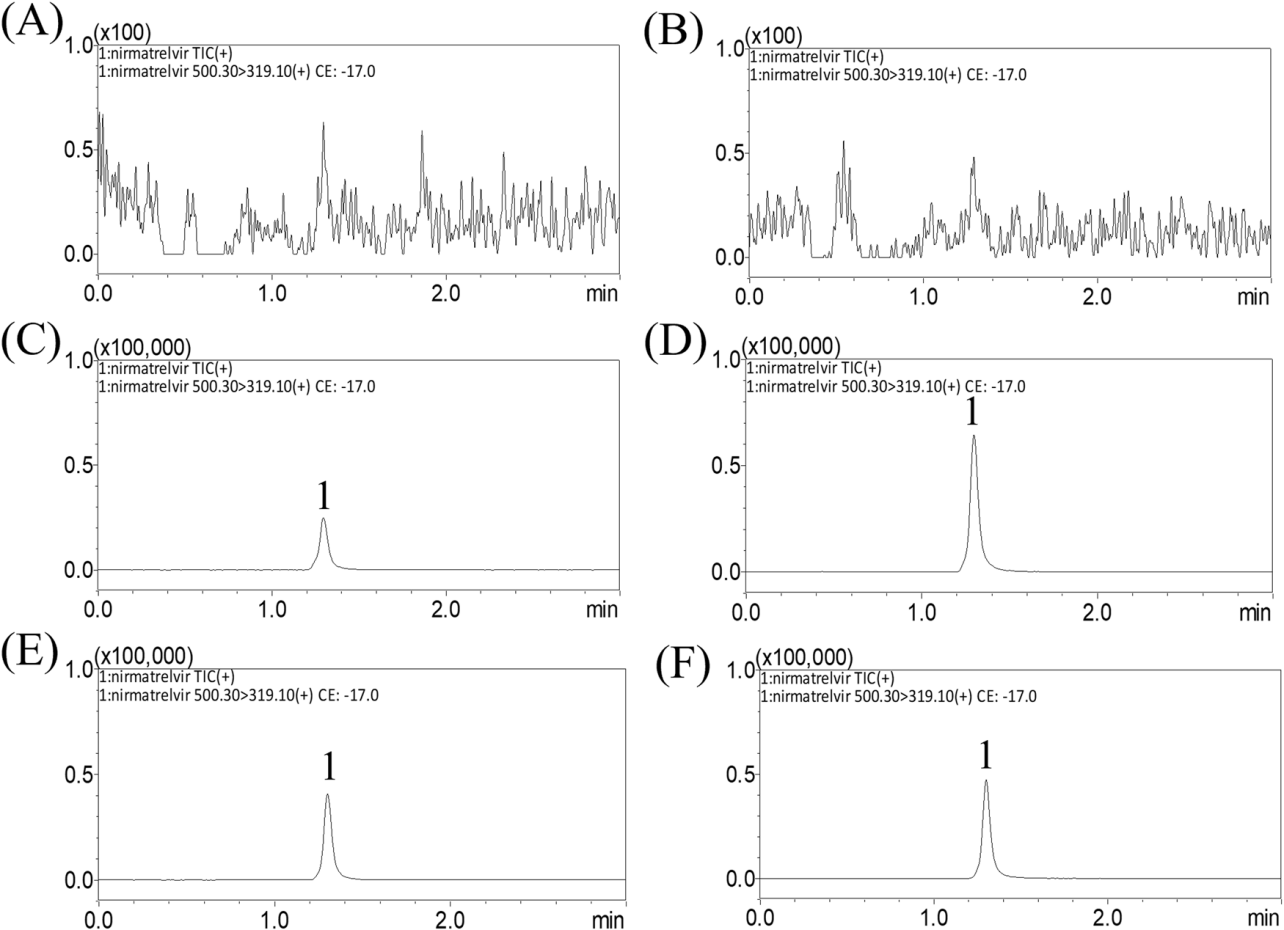
MRM chromatograms of nirmatrelvir in blood and brain. Representative MRM chromatograms of **A** blank blood dialysate, **B** blank brain dialysate, **C** blank blood dialysate spiked with nirmatrelvir (50 ng/mL), **D** blank brain dialysate spiked with nirmatrelvir (50 ng/mL), **E** blood dialysate sample containing nirmatrelvir (78.01 ng/mL), and **F** brain dialysate sample containing nirmatrelvir (36.36 ng/mL) collected 60 min after nirmatrelvir administration (15 mg/kg, i.v.); peak 1 = nirmatrelvir.

A validated UHPLC‒MS/MS system was developed to analyze nirmatrelvir in dialysate blood, conceptus, and brain. The quantitative determination limit of nirmatrelvir in rat dialysates was 2 ng/mL. Because nirmatrelvir has high hydrophobicity, the reverse C18 column was selected to help retain the compound, and the retention time was 1.30 min for nirmatrelvir (Supplementary Fig. 2). Representative multiple reaction monitoring (MRM) chromatograms of the blank matrix revealed that there was no interference of endogenous components during peak elution of dialysates of maternal blood, placenta, fetus, and amniotic fluid (Fig. 1). Similarly, in the BBB assay, there was no interference from endogenous components during the peak elution of dialysate from brain samples (Fig. 2). The UHPLC spectrum showed that in this optimized analysis method, there was no background noise interference, which means that the method had excellent selectivity. The results of the method validation are presented in Supplementary Tables 1–8.

### Method validation

The precision and accuracy of the analysis of nirmatrelvir spiked in blank dialysate from each tissue from female and male rats were tested in five replicates at the lower limit of quantitation (LLOQ) and low, medium and high concentrations (2, 5, 50, and 500 ng/mL). In the intraday and interday analysis, the precision and accuracy of the LLOQ were within the specified limits (less than ±20%), and the low, medium, and high concentrations were all within the specified limits (less than ±15%), which means that this analytical method has good repeatability and reproducibility (Tables S1 and S2).

The stability was determined in an autosampler at 4 °C for 6 h, on a bench-top at room temperature for 6 h, after freeze–thaw at −20 °C for three cycles, and long-term at −20 °C for a month (Tables S3 and S4). All the following stabilities were tested in three replicates at low and high concentrations, and the results are shown in Tables S3 and S4. There was no significant difference between nirmatrelvir prepared from different tissues of blank dialysate among the stability groups. However, it can be observed in Tables S3 and S4 that nirmatrelvir was slightly degraded under long-term storage, especially in blood dialysate samples. Therefore, samples need to be analyzed quickly, and when preparing samples, it is best to keep them in a low-temperature environment. In the results of the analysis of repeated freezing–thawing, it can be found that the concentration of drug in each tissue had a slight increase, which means that during the operation of the experiment, it is necessary to avoid repeated thawing of the sample to keep the concentration of the drug in the sample stable. In the investigation of each situation, the samples showed excellent stability for each tissue.

The matrix effect (% ME) in each tissue was calculated and is presented in Tables S5 and Table S6. According to the references of the matrix effect, the closer ME (%) is to 100%, the less the signal of the compound is interfered with by the matrix. When ME (%) <100%, the matrix will inhibit the signal intensity of the compound; conversely, when ME (%) >100%, the signal intensity will be enhanced. The matrix effect we observed in maternal blood was the largest, while in the placenta, fetus and amniotic fluid, there was a lower matrix effect (Table S5). Additionally, the matrix effect of the BBB group was the largest in blood, while in brain tissue, there was a lower matrix effect (Table S6). The results show that this analysis method has good analytical results in placenta, fetus, amniotic fluid and brain tissue. To ensure the consistency of the analysis conditions in the experiment, we strive to optimize the conditions to minimize the matrix effect in blood.

### Animal experiment and pharmacokinetic study

Following the ARRIVE (Animal Research: Reporting of In Vivo Experiments) guidelines, microdialysis provides the advantages of Replacement, Refinement and Reduction of Animals in Research. Compared to conventional sampling methods, microdialysis allows continuous sampling of extracellular fluid without loss of fluid and, therefore, minimizes the disturbance of biological homeostasis in vivo^34^.

### Pharmacokinetics of nirmatrelvir in pregnant rat blood

The compartmental model was established according to the minimum values of the Akaike information criterion (AIC) in maternal blood. In the two-compartment model, the pharmacokinetic equations of nirmatrelvir were *C*_u_ = 124e^−0.12*t*^ + 32.3e^−0.02*t*^ and *C*_u_ = 142.9e^−0.13*t*^ + 28.38e^−0.01*t*^ in the administration of nirmatrelvir (15 mg/kg, i.v.) alone and nirmatrelvir (15 mg/kg, i.v.) plus ritonavir (7 mg/kg, i.v.), respectively. Here, *C*_u_ and *t* represent the protein-unbound concentration of nirmatrelvir and time, respectively. The area under the curve (AUC) values of nirmatrelvir in maternal blood increased by approximately 2-fold compared to the administration of nirmatrelvir alone and combined with ritonavir, respectively (Table 1). The maximum concentrations (*C*_max_) of nirmatrelvir in maternal blood were 156.4 ± 23.97 and 171.4 ± 52.32 μg/mL, respectively (Table 1).

**Table 1:** Pharmacokinetic parameters of nirmatrelvir in maternal blood, placenta, fetus, and amniotic fluid after nirmatrelvir (15 mg/kg, i.v.) administration compared with nirmatrelvir (15 mg/kg, i.v.) plus ritonavir (7 mg/kg, i.v.) administration in pregnant rats.

Parameter	Nirmatrelvir (15 mg/kg, i.v.) (*n* = 6)					Nirmatrelvir (15 mg/kg, i.v.) plus ritonavir (7 mg/kg, i.v.) (*n* = 6)						
	Maternal blood		Placenta	Fetus	Amniotic fluid	Maternal blood	Placenta			Fetus		Amniotic fluid
AUC_last_ (min μg/mL)	3130 ± 278.2	480.5 ± 71.5^a^		169.7 ± 32.34^a^	284.6 ± 52.6^a^	7227 ± 557.7^ab^	1083 ± 202^ab^			343.5 ± 107.4^a^	1030 ± 267^a^	
*C*_max_ (μg/mL)	156.4 ± 23.97		3.56 ± 0.32^a^	0.96 ± 0.19^a^	1.84 ± 0.49^a^	171.4 ± 52.32		4.4 ± 0.82^a^		1.72 ± 0.6^a^	6.59 ± 1.84^a^	
*t*_1/2_ (min)	15 ± 1		109 ± 20	182 ± 60	167 ± 41	40 ± 7^b^		256 ± 39^b^		822 ± 546	241 ± 105	
*T*_max_ (min)	–		40 ± 5	70 ± 9	63 ± 22	–		132 ± 32^b^	236 ± 22^b^		216 ± 42^b^	
CL (mL/min/kg)	5 ± 0.4		–	–	–	2.14 ± 0.15^b^		–	–		–	
MRT (min)	48 ± 5		152 ± 23	308 ± 86	290 ± 55	193 ± 20^b^		415 ± 38^b^	1268 ± 784		457 ± 126	
AUC_tissue_/AUC_blood_	–		0.15 ± 0.03	0.06 ± 0.01	0.12 ± 0.03	–		0.13 ± 0.02	0.06 ± 0.02		0.20 ± 0.07	
AUC_combined_/AUC_alone_	–		–	–	–	2.31		2.25	2.03		3.61	

The data are expressed as the mean ± S.E.M. (*n* = 6). AUC_tissue_/AUC_blood_ represents the maternal blood-to-tissue transfer ratio. The combination ratio (AUC_combined_/AUC_alone_) represents the AUC ratio of nirmatrelvir (15 mg/kg, i.v.) plus ritonavir (7 mg/kg, i.v.) combined administration and nirmatrelvir (15 mg/kg, i.v.) alone administration.

*AUC* area under the concentration versus time curve, *C*_*max*_ the maximum concentration, *t*_*1/2*_ elimination half-life, *T*_*max*_ the time to reach the *C*_max_, *CL* clearance, *MRT* mean residence time.

^a^*p* < 0.05 compared with maternal blood in the nirmatrelvir (15 mg/kg, i.v.) group by one-way ANOVA with Tukey’s HSD post hoc test.

^b^*p* < 0.05 compared with the nirmatrelvir (15 mg/kg, i.v.) only group in the same tissue by independent *t* test.

### Transplacental transfer of nirmatrelvir

**Fig. 3:**
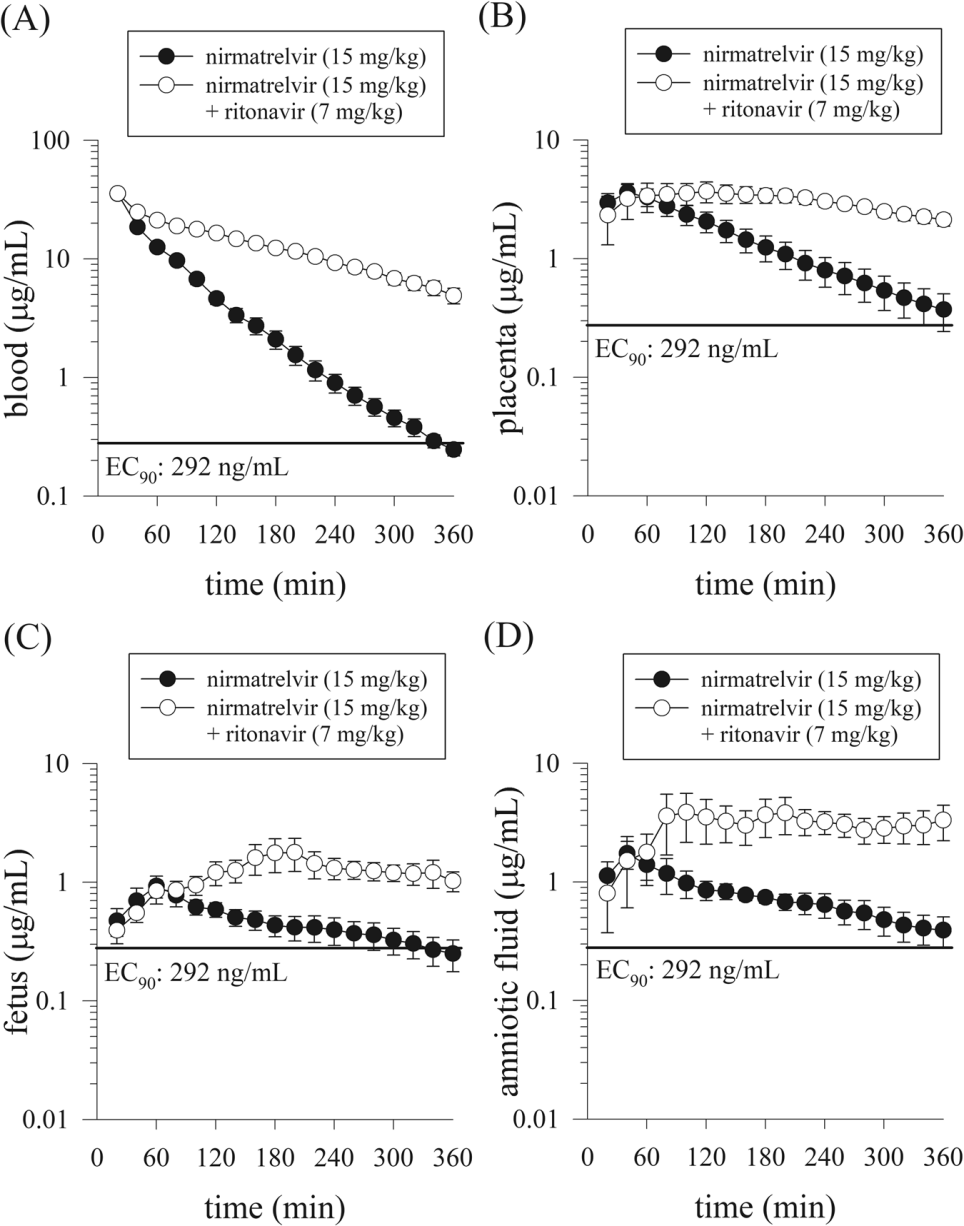
Concentration–time curves of nirmatrelvir in the maternal blood and conceptus. Concentration–time curves of protein-unbound nirmatrelvir in the rat after nirmatrelvir (15 mg/kg, i.v.) and nirmatrelvir (15 mg/kg, i.v.) plus ritonavir (7 mg/kg, i.v.) administration (*n* = 6). **A** Maternal blood, **B** placenta, **C** fetus, **D** amniotic fluid. The line is an EC_90_ (total EC_90_ = 292 ng/mL, unbound EC_90_ = 90.5 ng/mL, 181 nM)^12^; EC_90_ concentration at which 90% inhibition of viral replication is observed.

Due to the path through the biological barrier, the conceptus tissues (fetus, placenta, and amniotic fluid) were calculated by a noncompartment model. The *C*_max_ and AUC of nirmatrelvir in maternal blood, placenta, fetus, and amniotic fluid were increased significantly after the combined administration of nirmatrelvir (15 mg/kg, i.v.) plus ritonavir (7 mg/kg, i.v.), which suggested that ritonavir (7 mg/kg, i.v.) significantly enhanced nirmatrelvir levels in the maternal blood and conceptus tissues (Fig. 3 and Table 1).

The combination ratio (AUC_combined_/AUC_alone_) represents the AUC ratio of the combined administration of nirmatrelvir (15 mg/kg, i.v.) plus ritonavir (7 mg/kg, i.v.) and the administration of nirmatrelvir (15 mg/kg, i.v.) alone. The more than twofold range (approximately 2.03–3.61) of the combination ratio in maternal blood, placenta, fetus and amniotic fluid suggests a synergistic pharmacokinetic behavior and parallel distribution between the groups of nirmatrelvir plus ritonavir and nirmatrelvir alone (Table 1).

### Pharmacokinetics of nirmatrelvir in male rats

**Fig. 4:**
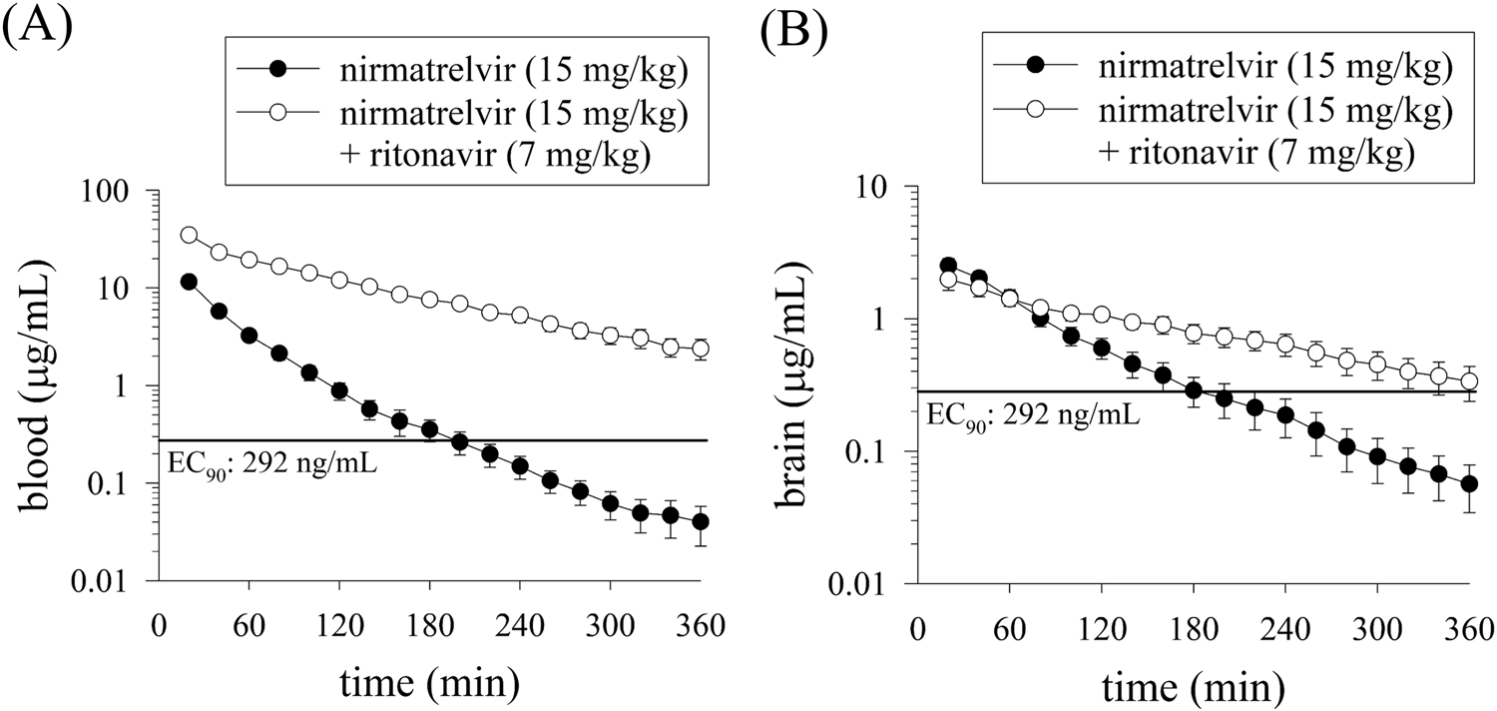
Concentration–time curves of nirmatrelvir in the blood and brain. Concentration–time curves of protein-unbound nirmatrelvir in the male rat blood (**A**) and brain (**B**) after nirmatrelvir (15 mg/kg, i.v.) and nirmatrelvir (15 mg/kg, i.v.) plus ritonavir (7 mg/kg, i.v.) administration (*n* = 6). The line is EC_90_ (total EC_90_ = 292 ng/mL, unbound EC_90_ = 90.5 ng/mL, 181 nM); EC_90_ concentration at which 90% inhibition of viral replication is observed^12^.

The compartmental model was utilized to evaluate the pharmacokinetics of nirmatrelvir in blood dialysates of male rats. The pharmacokinetic equations were *C*_u_ = 19.2e^−0.05*t*^ + 7.3e^−0.02*t*^ and *C*_u_ = 67.76e^−0.08*t*^ + 26.74e^−0.01*t*^ after nirmatrelvir (15 mg/kg, i.v.) alone and nirmatrelvir (15 mg/kg, i.v.) plus ritonavir (7 mg/kg, i.v.), respectively. The *C*_max_ of nirmatrelvir in blood after nirmatrelvir (15 mg/kg, i.v.) plus ritonavir (7 mg/kg, i.v.) were found to be approximately fivefold higher than those in the nirmatrelvir (15 mg/kg, i.v.) alone group (Fig. 4A and Table 2).

**Table 2:** Pharmacokinetic parameters of nirmatrelvir in the blood and brain after nirmatrelvir (15 mg/kg, i.v.) administration compared with nirmatrelvir (15 mg/kg, i.v.) plus ritonavir (7 mg/kg, i.v.) administration in male rats and nonpregnant female rats.

Parameter	Nirmatrelvir (15 mg/kg, i.v.) (*n* = 6)		Nirmatrelvir (15 mg/kg, i.v.) plus ritonavir (7 mg/kg, i.v.) (*n* = 6)		
	Blood	Brain	Blood	Brain	
Male					
AUC_last_ (min μg/mL)	772.4 ± 76.62	212.4 ± 33.06	4830 ± 274.4^a^		311.3 ± 42.33
* C*_max_ (μg/mL)	26.48 ± 4.45	2.52 ± 0.32	94.49 ± 27.65^a^		2.02 ± 0.35
* t*_1/2_ (min)	23 ± 4	66 ± 7	50 ± 9		123 ± 22^a^
*T*_max_ (min)	–	20 ± 0	–		27 ± 4
CL (mL/min/kg)	21.04 ± 2.80	–	3.16 ± 0.18^a^		–
MRT (min)	43 ± 5	92 ± 9	139 ± 26^a^		204 ± 29^a^
AUC_brain_/AUC_blood_	–	0.26 ± 0.03	–		0.06 ± 0.01^a^
AUC_combined_/AUC_alone_	–	–	6.25 ± 0.35		1.47 ± 0.19
Nonpregnant female					
AUC_last_ (min μg/mL)	1011 ± 97.72	187.5 ± 18.88	4163 ± 305.6^a^		316.6 ± 41.5^a^
*C*_max_ (μg/mL)	35.57 ± 2.11	2.42 ± 0.46	107.2 ± 7.40^a^		2.33 ± 0.39
*t*_1/2_ (min)	20 ± 2	65 ± 7	25 ± 1		64 ± 12
*T*_max_ (min)	–	20 ± 0	–		36 ± 11
CL (mL/min/kg)	15.97 ± 2.02	–	3.73 ± 0.29^a^		–
MRT (min)	40 ± 3	93 ± 5	79 ± 3^a^		132 ± 8^a^
AUC_brain_/AUC_blood_	–	0.21 ± 0.04	–		0.08 ± 0.01^a^
AUC_combined_/AUC_alone_	–	–	4.12 ± 0.30		1.68 ± 0.22

The data are expressed as the mean ± S.E.M. (*n* = 6). AUC_brain_/AUC_blood_ represents the blood-to-brain transfer ratio. AUC_combined_/AUC_alone_ represents the nirmatrelvir (15 mg/kg, i.v.) alone administration compared with the nirmatrelvir (15 mg/kg, i.v.) plus ritonavir (7 mg/kg, i.v.) combined administration ratio.

*AUC* area under the concentration versus time curve, *C*_*max*_ the maximum concentration, *t*_*1/2*_ elimination half-life, *T*_*max*_ the time to reach the *C*_max_, *CL* clearance, *MRT* mean residence time.

^a^*p* < 0.05 compared with the group treated with nirmatrelvir (15 mg/kg, i.v.) alone in the same tissue by independent *t* test.

### BBB transfer of nirmatrelvir in male rats

This study indicates a sixfold and 1.5-fold increase in AUC values in the blood and brain after the administration of nirmatrelvir (15 mg/kg, i.v.) and nirmatrelvir (15 mg/kg, i.v.) plus ritonavir (7 mg/kg, i.v.), respectively. The same phenomenon was observed in the *C*_max_, indicating that ritonavir (7 mg/kg, i.v.) synergistically improved nirmatrelvir levels in the blood (Fig. 4A), and slightly increase in the brain (Fig. 4B and Table 2). These findings indicate that nirmatrelvir is distributed to the tissue by blood and that the combination of nirmatrelvir and ritonavir has a significant impact on its pharmacokinetics.

### Pharmacokinetics of nirmatrelvir in nonpregnant female rats

**Fig. 5:**
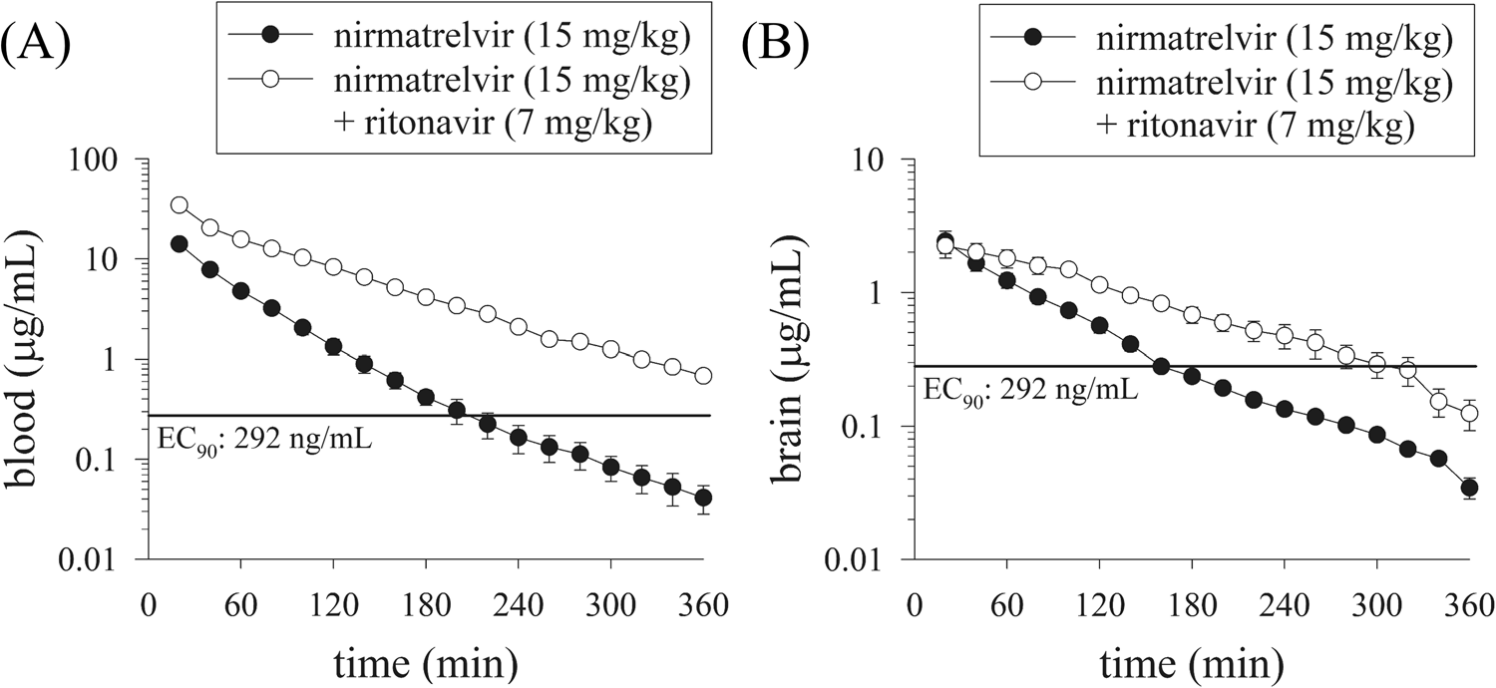
Concentration–time curves of nirmatrelvir in the blood and brain of nonpregnant female rats. Concentration–time curves of protein-unbound nirmatrelvir in the nonpregnant female rat blood (**A**) and brain (**B**) after nirmatrelvir (15 mg/kg, i.v.) and nirmatrelvir (15 mg/kg, i.v.) plus ritonavir (7 mg/kg, i.v.) administration (*n* = 6). The line is EC_90_ (total EC_90_ = 292 ng/mL, unbound EC_90_ = 90.5 ng/mL, 181 nM); EC_90_ concentration at which 90% inhibition of viral replication is observed^12^.

The pharmacokinetics of nirmatrelvir fit best to a two-compartment model with equations of *C*_u_ = 20.5e^−0.08*t*^ + 15.1e^−0.02*t*^ and *C*_u_ = 85.93e^−0.08*t*^ + 31.2e^−0.01*t*^ for nirmatrelvir (15 mg/kg, i.v.) administered alone and nirmatrelvir (15 mg/kg, i.v.) coadministered with ritonavir (7 mg/kg, i.v.), respectively in nonpregnant rats. Compared to nirmatrelvir administered alone, coadministration with ritonavir resulted in an approximately fourfold and threefold increase in the AUC and C_max_ of nirmatrelvir in the blood, respectively (Fig. 5A and Table 2). The time to reach the EC_90_ was extended from approximately 3 h to longer than 6 h, suggesting a synergistic effect in combination with ritonavir (Fig. 5A).

### BBB transfer of nirmatrelvir in nonpregnant female rats

Following the administration of nirmatrelvir (15 mg/kg, i.v.) plus ritonavir (7 mg/kg, i.v.), the AUC value in the brain tissue was about twofold increase in AUC within the brain tissue compared to nirmatrelvir (15 mg/kg, i.v.) alone (Fig. 5B and Table 2). Furthermore, *C*_max_ in brain tissue was not significantly different between the groups treated with nirmatrelvir alone and the combination of nirmatrelvir and ritonavir (Table 2). The time to reach the EC_90_ was extended from approximately 3 h to longer than 6 h, suggesting a synergistic effect in combination with ritonavir (Fig. 5A).

### Pharmacokinetics of nirmatrelvir in pregnant, nonpregnant female and male rats

**Fig. 6:**
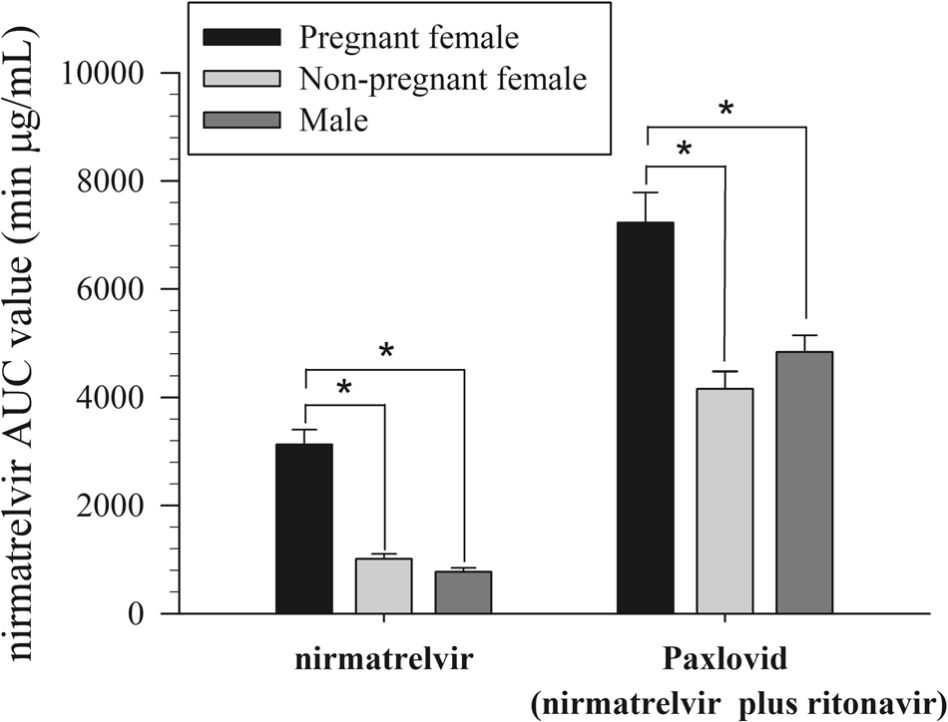
AUC bar chart of nirmatrelvir in blood for pregnant, nonpregnant, and male rats. Area under the curve (AUC) bar chart of protein-unbound nirmatrelvir in the blood after nirmatrelvir (15 mg/kg, i.v.) and nirmatrelvir (15 mg/kg, i.v.) plus ritonavir (7 mg/kg, i.v.) administration (*n* = 6) in the pregnant female rat, nonpregnant female rat and male rat. *p < 0.05 compared with pregnant female blood within group by one-way ANOVA with Tukey’s HSD post hoc test.

The AUC value of the pregnant group was higher than that of the other nonpregnant female and male rat groups and showed significant differences in the blood (Fig. 6). However, there was no significant difference in body drug concentration between nonpregnant female and male rats (Fig. 6). Compared to the gender effect of the nonpregnant female and male rat groups in the brain, the AUC values did not show a significant gender difference in the administration of nirmatrelvir alone or nirmatrelvir plus ritonavir. The trend in the blood-to-brain ratio was very similar to that of nonpregnant female and male rats, and there were no significant sex differences (Table 2).

## Discussion

Paxlovid is an oral drug, and the recommended dose of Paxlovid is 300 mg of nirmatrelvir plus 100 mg of ritonavir for humans. However, the aim of the study is to investigate biological barriers and to consider that the limitation of experimental technology is the simultaneous collection of a multisite biological sample using microdialysis. The experimental animal should be anesthetized during the experiment. Therefore, the route of intravenous administration was selected in this study. The major difference in pharmacokinetics between oral and intravenous is the first-pass effect for oral administration. To correct the route of oral and intravenous administration, we have adjusted the doses of nirmatrelvir and ritonavir by the oral bioavailability of nirmatrelvir and ritonavir, respectively. Furthermore, in this study, the dose was transferred from human to rat for drug administration. In Paxlovid, the daily dose of 300 mg of nirmatrelvir plus 100 mg of ritonavir was calculated by extrapolation to the animal dose through normalization to body surface area with the following formula: human equivalent dose (mg/kg) = animal dose (mg/kg) multiplied by *K*_m, animal_/*K*_m, human_. Here, *K*_m_ is estimated by dividing the average body weight (kg) of a species by its body surface area (m^2^); *K*_m, rat_ and *K*_m, human_ were 6 and 37 for rat and human, respectively^35^. The bioavailability of nirmatrelvir and ritonavir is approximately 50%^32^ and 80%^33^, respectively. Therefore, the doses of nirmatrelvir (15 mg/kg, i.v.) and nirmatrelvir (15 mg/kg, i.v.) plus ritonavir (7 mg/kg, i.v.) were used in the experiment.

A post hoc power analysis was performed to find the contrast administration between nirmatrelvir (15 mg/kg, i.v.) compared to the administration of nirmatrelvir (15 mg/kg, i.v.) plus ritonavir (7 mg/kg, i.v.) in the maternal blood and conceptus groups of pregnant rat groups (Fig. 3), male rat blood and brain (Fig. 4) and nonpregnant female rat blood and brain (Fig. 5). Based on the group sizes of 6, alpha 0.05, and a non-directional test, the power of nirmatrelvir in the two groups (with or without ritonavir) was higher than 80%. Furthermore, according to the research published by Arifin and Zahiruddin^36^, when using one-way ANOVA for group comparison between pregnant rats, nonpregnant rats, and male rats, the minimum sample size should be determined by the formula 10/*k* + 1, and the maximum sample size by the formula 20/*k* + 1, where *k* is the number of groups. Minimum *n* = 10/3 + 1 = 4.3, rounded up to 5 animals per group. Maximum *n* = 20/3 + 1 = 7.7, rounded down to 7 animals per group. Based on this reference, each group should ideally consist of 5–7 animals. Therefore, the experimental animal number 6 was used as the standard number of animals per group for experimentation in this study^36^.

Research in pregnant women with COVID-19 indicates that COVID-19 exists in the late pregnancy^19,37^. Although there are very few cases of vertical transmission of the placenta, vertical transmission of COVID-19 in pregnant women could not be ruled out. Facchetti et al. provided evidence for maternal–fetal vertical transmission of SARS-CoV-2, likely propagated by fetal mononuclear cells and confirmed by extensive virological and pathological investigations^38,39^. The safety and effectiveness of Paxlovid have been clinically studied in pregnant women, concluding that it helps relieve symptoms and no adverse effects on the fetus were found^18^. The reproductive safety toxicity of Paxlovid has been previously reported in the literature, without an effect on fertility or early embryonic development in male and female rats, and there was no evidence of severe developmental toxicity in the rabbit model^40^. In the zebrafish embryo model, nirmatrelvir does not affect survival rates or induce morphological defects at therapeutic doses in humans^41^. Taking into account drug delivery for pregnant patients, our previous reports have shown that remdesivir^26^, molnupiravir^16^, and their active circulating metabolites transfer to the placental barrier in an experimental animal model. In the present study, we showed that the transplacental barrier of nirmatrelvir to reach the conceptus (fetus, placenta, and amniotic fluid) and the level were higher than the EC_90_ (total EC_90_ = 292 ng/mL, unbound EC_90_ = 90.5 ng/mL, 181 nM)^12^ in regular dose of Paxlovid.

Infection with COVID-19 may not only lead to complications of encephalitis, but studies have also reported that the SARS-CoV-2 virus also has certain effects on the brain, potentially triggering long-term neurological problems. It has been confirmed that certain substances present in SARS-CoV-2 have the ability to cross the BBB and induce effects on the central nervous system. This finding establishes that specific components of the virus can infiltrate the brain, leading to neurological implications^42,43^. This means that in some cases, people infected with COVID-19 may face more serious health problems, especially those related to the nervous system^24^. Recently, a physiologically based LeiCNS-PK3.0 framework was applied and predicted that nirmatrelvir achieves effective concentrations against SARS-CoV-2 in human brain cells under the recommended dosing regimen. In contrast, two other antiviral drugs, remdesivir and molnupiravir, were unable to penetrate the BBB according to the model LeiCNS-PK3.0 of PBPK^44^. In this experiment, our results support the predictions of this PBPK model by measuring nirmatrelvir concentrations in the brain through in vivo microdialysis. The protein unbound nirmatrelvir concentrations in the blood and brain were all higher than the EC_90_^12^ in male and nonpregnant rats (Table 2).

Pregnancy is a dynamic state in which many physiologic and metabolic functions are altered. A previous report indicated that CYP3A activity is increased during pregnancy, and the apparent CYP3A activity was increased by 35-38% for midazolam^45^. Hebert et al. reported that the midazolam AUC_0 to infinity_ (ng h/mL) was 9.5 ± 4.3 and 17.9 ± 6.0 for pregnancy and postpartum, respectively, after midazolam administration (2 mg, p.o.) for healthy pregnant women, which suggested a 1.88-fold increase in CYP3A activity^46^. Compared to our data, there are two factors, protein binding and the enzyme CYP3A, involved in nonpregnancy and pregnancy. The AUC ratio of Paxlovid and nirmatrelvir alone (AUC_combined_/AUC_alone_) was 4.12 ± 0.30 for nonpregnant rats (Tables 1 and 2). However, the above AUC ratio was decreased to 2.31, which suggests that the CYP3A inhibition activity of ritonavir was compensated by the physiological pregnancy of increased CYP3A activity.

Gender differences in pharmacokinetics and pharmacodynamics have been discussed in the literature, such as hormonal changes during the menstrual cycle for females, protein binding in the blood, and the metabolism of gender-specific cytochrome P450 enzymes^47^. Studies have highlighted differences in clearance rates for various CYP3A4 substrates between sexes, indicating sex-related differences in drug metabolism^48^. Our experimental data compared the pharmacokinetics of nirmatrelvir alone and Paxlovid in male and nonpregnant rats. No significant differences in nirmatrelvir blood and brain performance were observed between nonpregnant female rats and male rats, both in single-dose and combined-dose settings (Table 2 and Fig. 6). These results demonstrated that there was no significant pharmacokinetic difference between sexes.

With respect to the construction of complexes for nirmatrelvir and ritonavir, if these two drugs form a complex, overcoming the challenge of molecular size will be a significant hurdle when dealing with biological barriers. Biological barriers are characterized by tight connections, typically allowing only compounds smaller than 500 Da to pass through^49^. It is suggested that ritonavir in the Paxlovid combination plays the main role of the CYP3A4 inhibitor in suppressing nirmatrelvir metabolism.

Based on the literature survey, there is limited information on CYP3A inhibitors that increase BBB permeability during pregnancy on PubMed. Regarding the BBB, current studies suggest that the expression of the CYP family in the brain is only approximately 10% of that in the liver^50,51^, which could exert a smaller influence on drug elimination. However, research indicates that CYP3A expression in the rat brain increases after ritonavir treatment with ritonavir (a CYP3A inhibitor)^52^. In our study, ritonavir, which serves as a CYP3A inhibitor, plays a similar role, leading to an increase in nirmatrelvir concentration in the blood. However, the interaction between nirmatrelvir and the BBB is not clear yet, necessitating further research on nirmatrelvir in the brain to gain a clearer understanding of the mechanism. The USFDA product label highlights that many drugs related to CYP3A have nirmatrelvir potential to influence the levels in the body. For example, antifungal drugs mentioned on the product label may lead to an increase in the concentration of nirmatrelvir/ritonavir in the body^15^.

Our data demonstrated that nirmatrelvir crosses biological barriers such as the BBB and the blood–placenta barrier to tissues. Ritonavir provides a synergistic effect and enhances the level of nirmatrelvir into biological barriers. However, one of the limitations of this study is that detailed mechanisms such as modulation in the biological barrier have not yet been investigated. The issue of the relationship between pregnancy and BBB permeability. The literature survey found that vascular endothelial growth factor (VEGF) and placental growth factor (PLGF) increase in the maternal circulation during pregnancy. An additional finding suggests that these factors can increase BBB permeability^53^. However, due to the limitation of surgical processes in combination with maternal blood and conceptus biological samples, we did not simultaneously collect brain dialysates with a stereotaxic instrument to facilitate brain surgery from pregnant rodents.

In our experiment, although we observed higher concentrations of nirmatrelvir in the bodies of pregnant rats compared to nonpregnant counterparts, nirmatrelvir toxicity has not been observed in animal models thus far. The safety and efficacy of Paxlovid have been clinically studied in pregnant women, revealing symptom relief without adverse effects on the fetus^18^. Previous literature reports on the reproductive safety toxicity of Paxlovid in animal models, including studies in male and female rats that did not show an impact on fertility or early embryonic development, and no evidence of severe developmental toxicity in rabbit models^40^. Furthermore, in the zebrafish embryo model, nirmatrelvir did not affect survival rates or induce morphological defects at therapeutic doses for humans^41^. Therefore, we lean toward following the indications provided on the product label for the administration of this medication.

In conclusion of the scientific findings of this article, (1) an in vivo multisite microdialysis system coupled to UHPLC-MS/MS system was developed to simultaneously monitor protein-unbound nirmatrelvir in maternal blood and conceptus in pregnant rats; (2) the pharmacokinetics and trans BBB have been shown to have no gender difference in the nonpregnant female and male rats; (3) the status of pregnant can reduce protein binding and enhance the protein-unbound form of nirmatrelvir; (4) the synergistic effect of ritonavir has been confirmed to boost nirmatrelvir levels in the biological samples for pregnant, nonpregnant female and male rats; (5) based on therapeutic concern, nirmatrelvir in the biological samples were all higher than the effective concentration 90 (EC90); (6) the main pharmacokinetic parameters such as AUC, *C*_max_, *T*_max_, and transportation ratios (AUC_tissue_/AUC_blood_; AUC_brain_/AUC_blood_; AUC_combined_/AUC_alone_) were analyzed. Our intention is to contribute to understanding and considerations surrounding the use of nirmatrelvir, particularly in the context of pregnancy and potential interactions with drugs metabolized in the brain.

## Supplementary information

Supplementary Information

## Data Availability

Experimental data are available upon reasonable request from the authors.
