# Supplementary material for: The anti-COVID-19 drug Paxlovid crosses biological barriers of the placenta and brain in rats
Source: Npj Viruses. 2024 Jan 24;2:4. doi: 10.1038/s44298-023-00013-1 (PMC11702666; doi:10.1038/s44298-023-00013-1)
Supplement: Supplementary file 1 — Supplementary Information [file 44298_2023_13_MOESM1_ESM.pdf]

Supplementary Information

# The anti-COVID-19 drug Paxlovid crosses biological barriers of the placenta and brain in rats

Wan-Hsin Lee[1], Chung-Kai Sun[1], Chun-Hao Chang[1], Muh-Hwa Yang[2], Tung-Hu Tsai[1,3,4,5]*

[1] Institute of Traditional Medicine, College of Medicine, National Yang Ming Chiao Tung University, Taipei 112, Taiwan

[2] Institute of Clinical Medicine, College of Medicine, National Yang Ming Chiao Tung University, Taipei 112, Taiwan

[3] Graduate Institute of Acupuncture Science, China Medical University, Taichung 404, Taiwan

[4] Department of Chemistry, National Sun Yat-Sen University, Kaohsiung 804, Taiwan

[5] School of Pharmacy, Kaohsiung Medical University, Kaohsiung 807, Taiwan

Shortened title: Biological barrier transfer of Paxlovid

------------------------------------

*Author for correspondence:
Tung-Hu Tsai, PhD,
Professor,
Institute of Traditional Medicine, College of Medicine, National Yang Ming Chiao Tung University, 155, Li-Nong Street Section 2, Taipei 112, Taiwan
Fax: (886-2) 2822 5044; Tel: (886-2) 2826 7115;
E-mail: thtsai@nycu.edu.tw

## S2. Materials and Methods

### S.2.1. UHPLC–MS/MS Conditions

The UHPLC–MS/MS system (Shimadzu LC–MS/MS 8030, Kyoto, Japan) used an Inertsil ODS-3 HP column (100 x 2.1 mm, particle size 3 μm, GL sciences, Japan) operated in a column oven at approximately 35°C for the entire experimental analysis. The UHPLC–MS/MS system consisted of a quadrupole mass spectrometer equipped with a positive mode electrospray ionization (ESI+) source that was used for nirmatrelvir analysis. Mobile phases A and B consisted of 0.1% formic acid in water (pH=2.6) and acetonitrile with isocratic elution at 40:60 (v/v), with a flow rate of 0.4 mL/min and an injection volume of 5 μL. The mass spectrometric conditions were set as follows: desolvation line temperature, 250°C; heat temperature, 400°C; interface voltage, 3.5 kV; nebulizing gas flow, 3.0 L/min; and drying gas flow, 15 L/min. Detection and quantification were performed using multiple reaction monitoring (MRM) for the parent-to-product ion transitions m/z 500.30 → 319.10 for nirmatrelvir (Supplemental Figure 1 and Supplementary Figure 2).

### S.2.2. Chemicals and Reagents

Nirmatrelvir was obtained from MedChemExpress (Monmouth Junction, NJ, USA). Ritonavir was purchased from Cayman Chemical Company (Ann Arbor, Michigan, USA). Dimethyl sulfoxide (DMSO), polyethylene glycol 400 (PEG400), heparin sodium salt, and urethane were purchased from Sigma–Aldrich Chemicals (St. Louis, MO, USA). Ethanol was acquired from ECHO Chemical Co., Ltd. (Miaoli County, Taiwan). Acetonitrile (mass grade) was acquired from J.T. Baker, Inc. (Phillipsburg, NJ). Triple deionized water from Millipore (Bedford, MA, USA) was used for all aqueous samples and mobile phase preparation. A standard stock solution of nirmatrelvir (1 mg/mL) was dissolved in ethanol and then stored at -20 °C for experimental use.

### S.2.3. Method Validation

The method validation, including the generation of the calibration curve; microdialysis probe recovery; assessments of the accuracy, precision, and stability of the animal samples; and matrix effect of each tissue, was based on the US FDA bioanalytical method validation guidelines.[1] Stock solutions of nirmatrelvir (1 mg/mL) were prepared in 10% DMSO in ethanol and stored at -20°C for experiments.

The calibration curve was prepared by dissolving nirmatrelvir (1 mg/mL) in 10% DMSO in ethanol from a stock solution and mixing the solution with blank rat dialysate at a series of dilution concentrations of 20, 50, 100, 500, 1000, 2500, and 5000 ng/mL. The concentration range of the calibration curve was approximately 2 to 500 ng/mL, and the coefficient of determination ($R^2$) value was higher than 0.995.

The accuracy and precision of standard samples were evaluated with the lower limit of quantitation (LLOQ) using low, medium, and high concentrations by analyzing them in five replicates on the same

day (intraday) and on five consecutive days (interday). Accuracy and precision involved calculating the difference between the observed concentration ($C_{obs}$) and the nominal concentration ($C_{nom}$). The accuracy (bias %) was calculated as [($C_{obs}$) – ($C_{nom}$)/$C_{nom}$] × 100%; the precision was calculated as the relative standard deviation, (RSD %) = [standard deviation (SD)/$C_{obs}$] × 100%. Analytical method validation and the RSD were all within acceptable limits of ± 15%, except at the LLOQ, which had to be within ± 20%.

To measure the stability, the stocks of nirmatrelvir were spiked into the blank dialysates of each tissue according to the following stages: autosampler, bench-top, freeze–thaw, and long-term stability. Data were collected using low (5 ng/mL) and high (250 ng/mL) concentrations of nirmatrelvir, and the samples that were freshly prepared were compared with the sample placed after the following four conditions. First, bench-top stability was conducted after the samples sat at room temperature for 6 hours. Second, the autosampler stability was determined by placing the samples in the autosampler of the machine at 4°C for 6 hours. Third, freeze–thaw stability was detected by freezing and thawing the analytes every 12 hours for three cycles. Finally, the long-term stability was determined by storing the analytes at -20°C for 1 month. Stability (%) = (the peak integration of the processed sample / the peak integration of the freshly prepared sample) × 100%.

At the same time, we also compared the matrix effect in each tissue to check whether the ions between different matrixes will affect the signal intensity of nirmatrelvir. Two sets of standard lines were prepared to evaluate the accuracy, precision, recovery and presence of matrix effects of the method. Prepare the first set of standard lines (Set 1) to evaluate the MS/MS response of nirmatrelvir standards spiked into neat solution. The second set (Set 2) was prepared in neat solution extracts and spiked after extraction.

Set 1: To prepare standard lines, ACD solution was used as a neat solution. For each sample, 45 µL of ACD solution was spiked with 5 µL of nirmatrelvir standard stock and transferred to a 1.5-mL centrifuge tube. The solutions were thoroughly mixed, transferred to 200-µL inserts and placed in vials. Finally, 5 µL of each solution was injected directly into the UHPLC–MS/MS system.

Set 2: For each sample in set 2, start by adding 45 µL of blank dialysate (this is an ACD solution that has been extracted in vivo) to a 1.5-mL centrifuge tube. Then, add 5 µL of nirmatrelvir standard stock solution to the same tube. Thoroughly mix the solution and transfer it to a 200-µL insert. Place the insert into a vial and ensure that it is securely capped. Finally, 5 µL of each solution was injected directly into the UHPLC–MS/MS system for analysis.

The formula of the matrix effect can be calculated by matrix effect (% ME) = (B/A) × 100%, where A is the peak area of the neat solution (ACD solution) spike stock standard as set 1 and B is the peak area

of the neat solution extracts (blank dialysate) of each tissue spike stock standard as set 2.[2]

The matrix effect is calculated in this way and can be referred to as the "absolute" matrix effect, comparing the signal response of a standard present in blank dialysate to that of a standard prepared directly in neat solution.

S.2.4. In vitro recovery of microdialysis probes

In this study, we used 3 types of probes, including blood, conceptus and brain probes. The difference between the analysis of these three probes is that different lengths of semipermeable membranes are used in verified tissues. The blood microdialysis probe was designed to be 11 mm, and the conceptus and brain tissue microdialysis probes were designed to be 6 mm. The probes are analyzed using a concentric silica capillary with a semipermeable dialysis membrane at the tip. The semipermeable membrane consisted of cellulose (Spectrum, New Brunswick, NJ, USA), and the molecular weight cutoff was 13,000 Da. The semipermeable membrane uses the concentration difference between the tissue and probe to detect the tissue drug concentration at successive time points. The recovery of microdialysis probes was evaluated by the following formula: true tissue drug concentration = observed drug concentration/recovery of probes. The recovery ($R_{dial}$) was calculated by comparing the analyte concentration in the dialysate ($C_{dial}$) and the nominal concentration ($C_{nom}$) with three replicated: $R_{dail}$ (%) = ($C_{dial}$/$C_{nom}$) × 100%.

S.2.5. Microdialysis experiment

The microdialysis instrument consisted of a microinjection pump (CMA/400; Solna, Sweden) and a microfraction collector (CMA/142). The laboratory-made microdialysis probe consisted of a concentric silica capillary and a semipermeable dialysis membrane (Spectrum, New Brunswick, NJ, USA) with a fiber diameter of 200 μm and a molecular weight cutoff of 13 kDa. The active lengths of the blood probe were 1.1 cm, and those of the conceptus and brain probes were 0.6 cm. The microinjection pump contained the perfusion fluid, which consisted of 3.5 mM citric acid, 7.5 mM sodium citrate, and 13.6 mM D-(+)-glucose (acid citrate dextrose; ACD solution), which was continuously infused at a flow rate of 2 μL/min; dialysates were collected every 20 minutes by a microfraction collector for 6 hr, and then the samples were stored at -20°C for analysis.

**S3. Results**

S.3.1. Method validation

The precision and accuracy of the analysis of nirmatrelvir spiked in blank dialysate from each tissue from female and male rats were tested in five replicates at the lower limit of quantitation (LLOQ) and low, medium and high concentrations (2, 5, 50, and 500 ng/mL). In the intraday and interday analysis, the precision and accuracy of the LLOQ were within the specified limits (less than ± 20%), and the low, medium, and high concentrations were all within the specified limits (less than ± 15%),

which means that this analytical method has good repeatability and reproducibility (Supplementary Table 1; Supplementary Table 2).

The stability was determined in an autosampler at 4℃ for six hours, on a bench-top at room temperature for six hours, after freeze−thaw at -20°C for three cycles, and long-term at -20°C for a month (Supplementary Table 3; Supplementary Table 4). All the following stabilities were tested in three replicates at low and high concentrations, and the results are shown in Supplementary Table 3 and Supplementary Table 4. There was no significant difference between nirmatrelvir prepared from different tissues of blank dialysate among the stability groups. However, it can be observed in Supplementary Table 3 and Supplementary Table 4 that nirmatrelvir was slightly degraded under long-term storage, especially in blood dialysate samples. Therefore, samples need to be analyzed quickly, and when preparing samples, it is best to keep them in a low-temperature environment. In the results of the analysis of repeated freezing-thawing, it can be found that the concentration of drug in each tissue had a slight increase, which means that during the operation of the experiment, it is necessary to avoid repeated thawing of the sample to keep the concentration of the drug in the sample stable. In the investigation of each situation, the samples showed excellent stability for each tissue.

The matrix effect (% ME) in each tissue was calculated and is presented in Supplementary Table 5 and Supplementary Table 6. According to the references of the matrix effect [1], the closer ME (%) is to 100%, the less the signal of the compound is interfered with by the matrix. When ME (%) <100%, the matrix will inhibit the signal intensity of the compound; conversely, when ME (%) >100%, the signal intensity will be enhanced. The matrix effect we observed in maternal blood was the largest, while in the placenta, fetus and amniotic fluid, there was a lower matrix effect (Supplementary Table 5). Additionally, the matrix effect of the blood–brain barrier group was the largest in blood, while in brain tissue, there was a lower matrix effect (Supplementary Table 6). The results show that this analysis method has good analytical results in placenta, fetus, amniotic fluid and brain tissue. To ensure the consistency of the analysis conditions in the experiment, we strive to optimize the conditions to minimize the matrix effect in blood.

S.3.2. In vitro recovery of the microdialysis probes

In vitro microdialysis is a technology that can be used to continuously detect the trend of drug concentration in the same tissue at different periods, which uses the probe as a sampling tool. Only the drugs that are not bound to the protein in the tissue can pass through the dialysis membrane and be collected using a concentration difference. In the blood-placenta barrier study, two different forms of probes were used for sampling: one was a blood probe placed in blood, and the other was a conceptus probe placed in the placenta, fetus, and amniotic fluid. The ratio of the level of nirmatrelvir that passed through the microdialysis probe membrane was calculated to confirm

whether nirmatrelvir can be stably dialyzed using concentration gradient differences at low, medium, and high concentrations. Because of the different lengths of probes, the in vitro recovery rate of nirmatrelvir was 4.90 ± 0.11 using the blood probe and 2.46 ± 0.11 using the conceptus probe (Supplementary Table 7). After statistical analysis using one-way ANOVA with Tukey's HSD *post hoc* test, there was no significant difference in the recovery rate between low, medium and high concentrations, which means that the sampling method was not different due to the concentration.

In the blood–brain barrier group, to evaluate the penetration of the dialysis membrane, in vitro recovery was performed to calculate the blood and brain microdialysis probes with active lengths of 11 and 6 mm. The in vitro recovery rates of nirmatrelvir in the blood and brain were 4.90 ± 0.05 and 2.45 ± 0.02, respectively (Supplementary Table 8). There was no significant difference within the nirmatrelvir concentration range in these two types of probes. The consistent recovery of microdialysis shows that microdialysis can be used as a stable sampling technique during the experiment.

**References**

1 US Food and Drug Administration. *Bioanalytical-Method-Validation-Guidance-for-Industry*, <https://www.fda.gov/files/drugs/published/Bioanalytical-Method-Validation-Guidance-for-Industry.pdf> (2018).

2 Matuszewski, B. K., Constanzer, M. & Chavez-Eng, C. Strategies for the assessment of matrix effect in quantitative bioanalytical methods based on HPLC− MS/MS. *Anal Chem.* **75**, 3019-3030 (2003). https://doi.org:10.1021/ac020361s

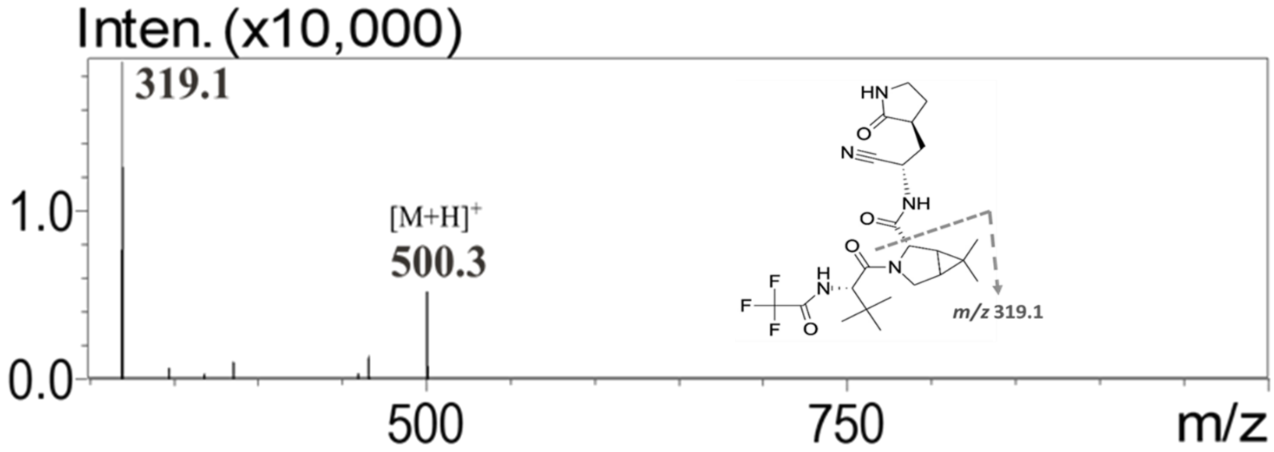


**Supplementary Figure 1.** MRM product ion mass spectra of nirmatrelvir at *m/z* 500.3 → 319.1.

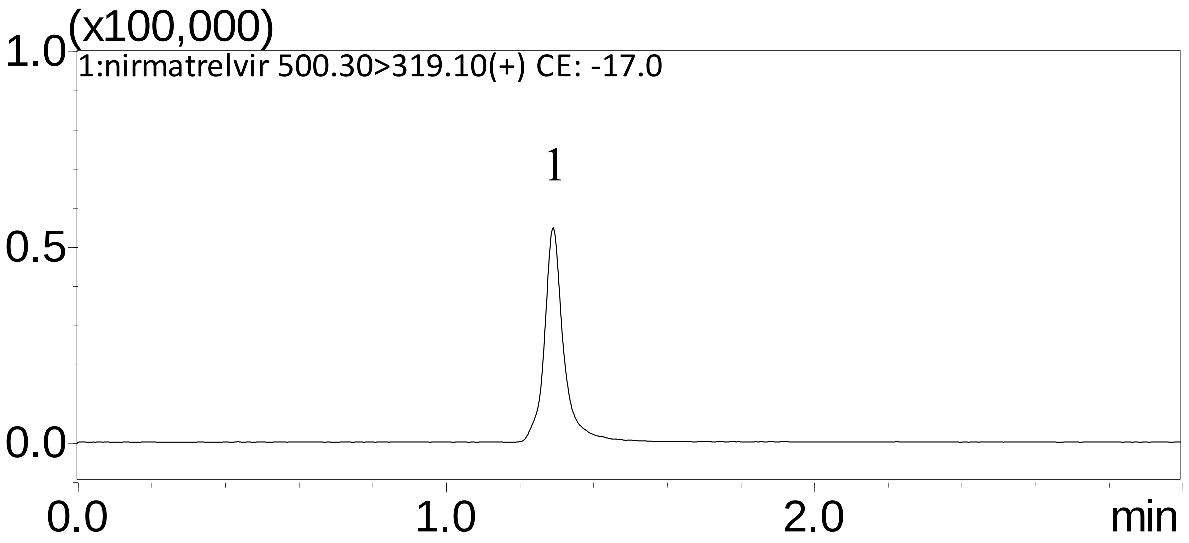


**Supplementary Figure 2.** Representative MRM chromatograms of nirmatrelvir; the retention time was 1.30 min. peak 1= nirmatrelvir

**Supplementary Table 1.** Intraday and interday precision (% RSD) and accuracy (% Bias) of the UHPLC–MS/MS method for the determination of nirmatrelvir in pregnant rat blood, placenta, fetus, and amniotic fluid dialysates.

| Nominal concentration (ng/mL) | Intraday | | | Interday | | |
|---|---|---|---|---|---|---|
| | Observed concentration (ng/mL) | Precision (% RSD) | Accuracy (% Bias) | Observed concentration (ng/mL) | Precision (% RSD) | Accuracy (% Bias) |
| Maternal blood | | | | | | |
| 2 | 2.29 ± 0.03 | 1.27% | 14.98% | 2.17 ± 0.17 | 7.97% | 13.75% |
| 5 | 5.04 ± 0.17 | 3.56% | 0.82% | 4.90 ± 0.25 | 5.03% | -2.08% |
| 50 | 50.75 ± 1.14 | 2.25% | 1.51% | 49.87 ± 0.67 | 1.34% | -0.26% |
| 500 | 500.4 ± 1.02 | 0.21% | 0.08% | 500.3 ± 0.20 | 0.04% | 0.05% |
| Placenta | | | | | | |
| 2 | 2.21 ± 0.07 | 3.59% | 10.59% | 1.91 ± 0.16 | 8.55% | -4.55% |
| 5 | 5.02 ± 0.18 | 3.73% | 0.32% | 5.00 ± 0.12 | 2.44% | 0.07% |
| 50 | 51.16 ± 2.5 | 4.91% | 2.34% | 50.16 ± 0.28 | 0.56% | 0.33% |
| 500 | 488.6 ± 5.22 | 1.07% | -2.30% | 500.2 ± 0.60 | 0.12% | 0.05% |
| Fetus | | | | | | |
| 2 | 2.16 ± 0.06 | 3.06% | 8.34% | 2.16 ± 0.12 | 5.38% | 7.77% |
| 5 | 4.97 ± 0.22 | 4.48% | -0.41% | 5.12 ± 0.28 | 5.57% | 2.43% |
| 50 | 49.68 ± 2.34 | 4.73% | -0.62% | 50.20 ± 0.87 | 1.74% | 0.39% |
| 500 | 475.2 ± 11.58 | 2.44% | -4.96% | 500.4 ± 0.20 | 0.04% | 0.08% |
| Amniotic fluid | | | | | | |
| 2 | 1.88 ± 0.07 | 3.78% | -5.81% | 2.01 ± 0.16 | 7.90% | 0.71% |
| 5 | 4.52 ± 0.15 | 3.41% | -9.47% | 5.12 ± 0.20 | 3.84% | 2.48% |
| 50 | 46.01 ± 2.31 | 5.05% | -7.98% | 50.18 ± 0.68 | 1.35% | 0.36% |
| 500 | 489.8 ± 10.18 | 2.08% | -2.02% | 500.9 ± 1.06 | 0.21% | 0.18% |

The data are expressed as the means ± SDs. (n = 5) Accuracy and precision involved calculating the difference between the observed concentration ($C_{obs}$) and the nominal concentration ($C_{nom}$). The accuracy (bias %) was calculated as $[(C_{obs}) - (C_{nom})/C_{nom}] \times 100\%$; the precision was calculated as the relative standard deviation, (RSD %) = $[\text{standard deviation (SD)}/C_{obs}] \times 100\%$.

**Supplementary Table 2.** Intraday and interday precision (% RSD) and accuracy (% Bias) of the UHPLC–MS/MS method for the determination of nirmatrelvir in male rats' blood and brain dialysates.

| Nominal concentration (ng/mL) | Intraday | | | Interday | | |
|---|---|---|---|---|---|---|
| | Observed concentration (ng/mL) | Precision (% RSD) | Accuracy (% Bias) | Observed concentration (ng/mL) | Precision (% RSD) | Accuracy (% Bias) |
| Blood | | | | | | |
| 2 | 2.32 ± 0.01 | 0.51% | 16.00% | 2.08 ± 0.16 | 7.49% | 4.25% |
| 5 | 5.36 ± 0.07 | 1.49% | 7.26% | 5.25 ± 0.37 | 7.03% | 5.05% |
| 50 | 49.48 ± 1.37 | 2.77% | -1.03% | 50.10 ± 0.99 | 1.98% | 0.21% |
| 500 | 510.1 ± 6.16 | 1.21% | 2.01% | 500.7 ± 0.92 | 0.18% | -0.16% |
| Brain | | | | | | |
| 2 | 2.28 ± 0.02 | 1.04% | 14.37% | 1.91 ± 0.13 | 6.78% | -4.46% |
| 5 | 5.13 ± 0.13 | 2.53% | 2.79% | 4.85 ± 0.19 | 3.86% | -2.81% |
| 50 | 50.46 ± 2.01 | 3.98% | 0.92% | 49.41 ± 1.81 | 3.67% | -1.16% |
| 500 | 512.8 ± 7.37 | 1.44% | 2.56% | 500.1 ± 0.38 | 0.08% | 0.02% |

The data are expressed as the means ± SDs. (n = 5) Accuracy and precision involved calculating the difference between the observed concentration ($C_{obs}$) and the nominal concentration ($C_{nom}$). The accuracy (bias %) was calculated as $[(C_{obs}) - (C_{nom})/C_{nom}] \times 100\%$; the precision was calculated as the relative standard deviation, (RSD %) = $[\text{standard deviation (SD)}/C_{obs}] \times 100\%$.

**Supplementary Table 3.** Stability of nirmatrelvir in the pregnant rat blood, placenta, fetus, and amniotic fluid dialysates.

| Nominal concentration (ng/mL) | Autosampler stability (%) | Bench-top stability (%) | Freeze–thaw stability (%) | Long-term stability (%) |
|---|---|---|---|---|
| Maternal blood | | | | |
| 5 | 102.98 ± 4.81 | 96.42 ± 2.53 | 101.21 ± 2.66 | 87.18 ± 0.87 |
| 250 | 98.97 ± 4.80 | 98.68 ± 1.18 | 105.98 ± 2.99 | 87.65 ± 1.83 |
| Placenta | | | | |
| 5 | 101.55 ± 6.56 | 100.37 ± 1.24 | 101.85 ± 4.27 | 97.38 ± 0.55 |
| 250 | 98.14 ± 3.84 | 98.87 ± 0.27 | 100.83 ± 0.95 | 88.97 ± 1.33 |
| Fetus | | | | |
| 5 | 95.10 ± 0.47 | 102.37 ± 2.51 | 107.56 ± 0.63 | 95.20 ± 1.21 |
| 250 | 97.12 ± 1.94 | 101.02 ± 0.48 | 98.02 ± 2.49 | 91.79 ± 1.56 |
| Amniotic fluid | | | | |
| 5 | 101.84 ± 1.89 | 104.82 ± 1.50 | 107.43 ± 5.37 | 97.21 ± 0.39 |
| 250 | 102.44 ± 2.35 | 98.60 ± 1.46 | 99.39 ± 4.32 | 93.43 ± 1.54 |

The data are expressed as the means ± SDs. (n = 3) Stability (%) = (the peak integration of the processed sample / the peak integration of the freshly prepared sample) × 100%.

**Supplementary Table 4.** Stability of nirmatrelvir in the blood and brain dialysates of male rats.

| Nominal concentration (ng/mL) | Autosampler stability (%) | Bench-top stability (%) | Freeze−thaw stability (%) | Long-term stability (%) |
|---|---|---|---|---|
| Blood | | | | |
| 5 | 95.56 ± 6.20 | 104.5 ± 1.73 | 100.3 ± 3.40 | 89.61 ± 1.00 |
| 250 | 94.23 ± 4.18 | 97.60 ± 0.73 | 88.57 ± 0.33 | 88.54 ± 1.07 |
| Brain | | | | |
| 5 | 92.51 ± 2.01 | 100.6 ± 0.37 | 99.58 ± 1.02 | 90.07 ± 2.77 |
| 250 | 93.59 ± 4.41 | 95.79 ± 0.24 | 92.01 ± 0.29 | 87.36 ± 2.58 |

The data are expressed as the means ± SDs. (n = 3) Stability (%) = (the peak integration of the processed sample / the peak integration of the freshly prepared sample) × 100%.

**Supplementary Table 5.** Matrix effect of nirmatrelvir in the pregnant rat blood, placenta, fetus, and amniotic fluid dialysates.

| Nominal concentration (ng/mL) | Matrix effect (%) | | | |
|---|---|---|---|---|
| | Maternal blood | Placenta | Fetus | Amniotic fluid |
| 5 | 9.30 ± 0.27 | 70.97 ± 1.70 | 87.03 ± 1.87 | 72.16 ± 1.44 |
| 250 | 9.35 ± 1.72 | 73.57 ± 1.26 | 86.87 ± 1.10 | 71.32 ± 1.95 |
| Average | 9.32 ± 0.03 | 72.27 ± 1.30 | 86.95 ± 0.08 | 71.74 ± 0.42 |

The data are expressed as the means ± SDs. (n = 3)

Matrix effect (% ME) = (B/A) × 100%, A= the peak area of neat solution (ACD solution) spike stock standard as set 1, B= the peak area of the neat solution extracts (blank dialysate) of each tissue spike stock standard as set 2.

**Supplementary Table 6.** Matrix effect of nirmatrelvir in male rat blood and brain dialysates.

| Nominal concentration (ng/mL) | Matrix effect (%) | |
|---|---|---|
| | blood | brain |
| 5 | 10.91 ± 0.30 | 58.69 ± 3.62 |
| 250 | 9.95 ± 0.20 | 57.49 ± 2.65 |
| Average | 10.43 ± 0.48 | 58.09 ± 0.60 |

The data are expressed as the means ± SDs. (n = 3)

Matrix effect (% ME) = (B/A) × 100%, A= the peak area of neat solution (ACD solution) spike stock standard as set 1, B= the peak area of the neat solution extracts (blank dialysate) of each tissue spike stock standard as set 2.

**Supplementary Table 7.** In vitro microdialysis recovery (%) of nirmatrelvir in the blood and conceptus probes using the ACD solution as the perfusion solution.

| Compound | Concentration (ng/mL) | Recovery (%) in blood probe | Recovery (%) in conceptus probe |
|---|---|---|---|
| Nirmatrelvir | 100 | 4.86 ± 0.10 | 2.41 ± 0.03 |
| | 500 | 4.92 ± 0.11 | 2.46 ± 0.18 |
| | 1000 | 4.93 ± 0.11 | 2.52 ± 0.12 |
| | Average | 4.90 ± 0.11 | 2.46 ± 0.11 |

The data are expressed as the means ± SDs. The recovery ($R_{dial}$) was calculated by comparing the analyte concentration in the dialysate ($C_{dial}$) and the nominal concentration ($C_{nom}$) with three replicated: $R_{dail}$ (%) = ($C_{dial}$/$C_{nom}$) × 100%.

**Supplementary Table 8.** In vitro microdialysis recovery (%) of nirmatrelvir in male rats' blood and brain probes using ACD solution as the perfusion solution.

| Compound | Concentration (ng/mL) | Recovery (%) in blood probe | Recovery (%) in brain probe |
|---|---|---|---|
| Nirmatrelvir | 100 | 4.86 ± 0.08 | 2.46 ± 0.06 |
| | 500 | 4.92 ± 0.04 | 2.43 ± 0.04 |
| | 1000 | 4.93 ± 0.02 | 2.47 ± 0.08 |
| | Average | 4.90 ± 0.05 | 2.45 ± 0.02 |

The data are expressed as the means ± SDs. The recovery ($R_{dial}$) was calculated by comparing the analyte concentration in the dialysate ($C_{dial}$) and the nominal concentration ($C_{nom}$) with three replicated: $R_{dail}$ (%) = ($C_{dial}$/$C_{nom}$) × 100%.
